# The Role of the Subthalamic Nucleus in Inhibitory Control of Oculomotor Behavior in Parkinson’s Disease

**DOI:** 10.1038/s41598-020-61572-4

**Published:** 2020-03-25

**Authors:** Shahab Bakhtiari, Ayca Altinkaya, Christopher C. Pack, Abbas F. Sadikot

**Affiliations:** Department of Neurology and Neurosurgery, Montreal Neurological Institute, McGill University, Montreal, Canada

**Keywords:** Saccades, Human behaviour, Parkinson's disease

## Abstract

Inhibiting inappropriate actions in a context is an important part of the human cognitive repertoire, and deficiencies in this ability are common in neurological and psychiatric disorders. An anti-saccade is a simple oculomotor task that tests this ability by requiring inhibition of saccades to peripheral targets (pro-saccade) and producing voluntary eye movements toward the mirror position (anti-saccades). Previous studies provide evidence for a possible contribution from the basal ganglia in anti-saccade behavior, but the precise role of different components is still unclear. Parkinson’s disease patients with implanted deep brain stimulators (DBS) in subthalamic nucleus (STN) provide a unique opportunity to investigate the role of the STN in anti-saccade behavior. Previous attempts to show the effect of STN DBS on anti-saccades have produced conflicting observations. For example, the effect of STN DBS on anti-saccade error rate is not yet clear. Part of this inconsistency may be related to differences in dopaminergic states in different studies. Here, we tested Parkinson’s disease patients on anti- and pro-saccade tasks ON and OFF STN DBS, in ON and OFF dopaminergic medication states. First, STN DBS increases anti-saccade error rate while patients are OFF dopamine replacement therapy. Second, dopamine replacement therapy and STN DBS interact: L-dopa reduces the effect of STN DBS on anti-saccade error rate. Third, STN DBS induces different effects on pro- and anti-saccades in different patients. These observations provide evidence for an important role for the STN in the circuitry underlying context-dependent modulation of visuomotor action selection.

## Introduction

Neuromodulation or Deep Brain Stimulation (DBS) of several subcortical areas significantly improves motor function in Parkinson’s disease (PD)^1,2^. Stimulation of the subthalamic nucleus (STN) improves tremor, drug-induced dyskinesias and motor fluctuations, and may also modulate aspects of executive cognition or emotion^3–6^. However, unlike its motor outcome, the effect of subthalamic DBS on higher order executive functions remains uncertain.

An important component of executive function is inhibitory control: the ability to suppress an impulsive response to external events due to an inappropriate context^7^. Dysfunction of inhibitory control is involved in several psychiatric conditions, such as mood disorders, disorders of thought, and addiction^8,9^. In PD, a deficiency of inhibitory control has been reported in different experimental tasks, such as the stop signal task^10^, and the Stroop task^11^, and impulse control may be worsened by therapies in some patients^12,13^.

Eye movements can be measured precisely and objectively (with 1 ms temporal resolution and approximately 0.5 degree spatial resolution) by using state-of-the-art eye-tracking technologies. This advance has made eye movements a popular readout behavior for probing different aspects of cognition with potential applications in clinical assessments^14^. One well-defined and widely used task for evaluating inhibitory control in humans^15–17^ and non-human primates^18–21^ is the oculomotor anti-saccade task. In anti-saccade behavior, unlike pro-saccades, the subject is asked to look away from a target that appears in the peripheral visual field. Inability to suppress the reflexive pro-saccade in the anti-saccade task indicates a deficiency in inhibitory control. Previous work indicates that PD patients generate a larger number of erroneous pro-saccades compared to healthy controls^22^, suggesting that the anti-saccade task may be used to understand how different parts of the basal ganglia network may participate in inhibitory control. The subthalamic nucleus is an important part of the basal ganglia network involved in inhibitory control of a wide variety of sensorimotor, visuomotor and cognitive behaviors^23^. PD patients with therapeutic implants placed in the subthalamic nucleus may therefore serve as an important model for understanding the role of the STN in the basal ganglia network that modulates anti-saccade performance. Evaluation of anti-saccade performance during STN DBS may also serve as a well-defined measure of impulsive behaviors that are increasingly recognized in PD patients, and that are modulated by both L-Dopa and STN DBS^24–27^.

Previous attempts to measure the effect of STN DBS on anti-saccade behavior in PD patients have resulted in variable observations. In two studies, PD patients were tested on and off STN DBS for the anti-saccade task while they were on their therapeutic levodopa (L-dopa) dose^28,29^. Both studies reported that STN stimulation does not significantly alter the error rate or latency of anti-saccades. In contrast, a recent study that tested patients while off L-dopa and equivalents^30^ suggests that STN DBS increases anti-saccade error rate without changing the latency of correct anti-saccades. The variable results of these three studies calls for a more controlled protocol to examine the effect of STN DBS on anti-saccade behavior. Specifically, since the major difference between the three study designs is the L-dopa state, it is important to evaluate the effect of STN DBS both during the on- and off-medication conditions. We proposed that an interaction between the on-medication state and subthalamic stimulation may potentially explain the conflicting results of previous studies. We measured anti-saccade behavior in a group of PD patients during all four possible conditions of stimulation and medication. Our results show that STN DBS increases anti-saccade error rate. Furthermore, this effect was larger when the patients were OFF L-dopa. In the ON L-dopa condition, the effect of STN DBS on anti-saccade error rate is less pronounced. Therefore, our data supports a complex interaction between stimulation and dopaminergic effects. The subthalamic nucleus appears to be an important part of the circuitry allowing generation of inhibitory anti-saccades in PD. Subthalamic stimulation, which allows for significant improvement in motor function in PD, appears to interfere with certain frontal inhibitory mechanisms likely generated by the oculomotor loop of the basal ganglia that targets the dorsolateral prefrontal cortex.

## Methods

### Subjects

This study was conducted at the Montreal Neurological Institute, and was reviewed and approved by McGill University Health Centre Institutional Review Board. All the methods were performed in accordance with approved guidelines. 10 PD patients (age $61.2\pm 5.95$ years; 3 females; 7 patients at Hoehn-Yahr Stage 3 and 3 patients at Stage 4) who had undergone STN DBS surgery were recruited for this study. All patients provided written informed consent before participating in this study.

### Procedure

**Figure 1:**
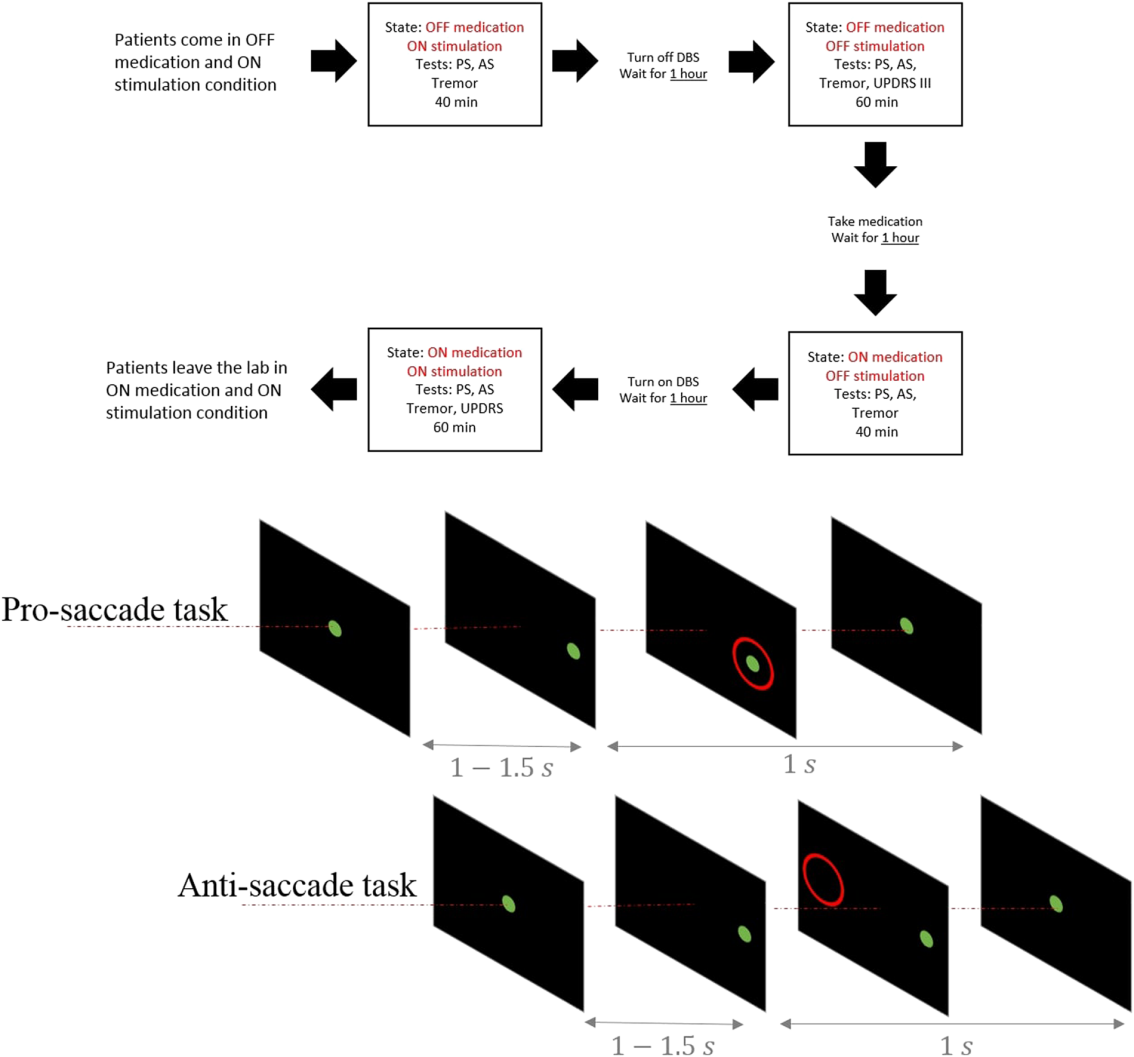
Experiment paradigm and the eye movement task. Top: Each box shows one of the test conditions. Bottom: The schematic figure shows the pro-saccade (top row) and the anti-saccade (bottom row) tasks.

Patients were tested in four different combinations of L-dopa or subthalamic stimulation states: (1) OFF L-dopa – ON stimulation, (2) OFF L-dopa – OFF stimulation, (3) ON L-dopa – OFF stimulation, and (4) ON L-dopa – ON stimulation (Fig. 1a). In each condition, patients were asked to participate in the same series of eye movement tests. Arm tremor was also measured using accelerometers as an index of L-Dopa or STN DBS related effects. Part III of the United Parkinson’s Disease Rating Scale (UPDRS) was performed at the end of the second phase (OFF L-dopa – OFF stimulation) and the fourth phase (ON L-dopa – ON stimulation). Patients were given a 45-60 minute break between testing in different conditions. The breaks relieved fatigue, and also assured a stable state as patients transitioned between therapeutic conditions. DBS parameters and morning medication doses used in the experiment were identical to those established as therapeutic over time by the patients’ clinicians.

Participants came to the lab early in the morning with their DBS ON, after stopping L-Dopa for about 12 hours. Patients first received a general introduction to different steps of the protocol that covered the whole day. They were then instructed on the eye movement tasks and went through the first block of tests in the OFF L-dopa – ON stimulation condition. After the first condition, DBS was turned off, and patients took a 45-60 minute break. Following the break, the same tests were repeated for the OFF L-dopa – OFF stimulation condition. At the end of this condition, patients took their regular dose of L-dopa (Table 1), and after the break period patients were again tested with the same series of tasks in the ON L-dopa – OFF stimulation condition. The DBS was then turned back on, and after the break patients were tested in the ON L-dopa – ON stimulation condition.

**Table 1:** Patients’ demographics.

ID	Age	Sex	Years from diagnosis	LED (mg)	UPDRS III DBS OFF	UPDRS III DBS ON
1	55	F	10	100	65	37
2	70	M	8	133	45	31
3	63	M	17	100	44	30
4	57	F	19	150	54	10
5	55	M	9	100	13	3
6	70	M	17	200	59	35
7	61	M	9	100	56	35
8	54	F	18	266	23	11
9	62	M	7	200	44	29
10	65	M	12	133	21	16

**LED:** L-dopa equivalent dose^66^ that was given to the patients at the time of the test.

### Tremor measurement

Participants were asked to wear an accelerometer (GENEActiv, Activinsights Ltd., Kimbolton, UK) on both wrists. They were then seated on a chair with arms supported with a custom-made arm support. The tremor was measured at each arm at rest during three 15 second trials with eyes open, and with a 30 second rest between trials when patients were encouraged to move their arms.

The accelerometer data was first trend corrected and then preprocessed with Principle Component Analysis (PCA). The first principle component was then used for further analysis. Multi-tapered power spectrum was calculated from the preprocessed data, and the power amplitude for the range of 3–6 Hz was normalized and used as a measure of tremor amplitude.

### Oculomotor tasks

Patients were seated in front of an OLED TV (LG 55EA9800, 122.7 cm × 79.86 cm) at a viewing distance of 57 cm. Chin and forehead rests were used to stabilize their heads and to maximize the accuracy of eye tracking.

Eye position was recorded using a desk-mounted EyeLink 1000 eye tracker (SR Research) at 1000 Hz. Recordings were monocular (right eye only), and the data were analyzed offline. The eye movement task included two blocks of pro-saccades (60 trials per block) and three blocks of anti-saccades (40 trials per block). The task started with the blocks of pro-saccades and finished with the anti-saccades. Patients had a one minute break between the blocks, and a two minute break between pro-saccade and anti-saccade trials. For both tasks, the fixation target, a green circle subtending 1.5 visual degrees (luminance $55.2\,cd/{m}^{2})$, appeared on the center of the screen at the beginning of each trial. After 0.5 to 1.5 seconds, it jumped to a 10 degree visual angle on the right or left side of the fixation point. The target then stayed on the screen for one second and jumped back to the central position which signaled the beginning of next trial.

In the pro-saccade task, patients were asked to look at the peripheral target as quickly and as accurately as possible. In the anti-saccade task, with the same stimulus, the patients were asked to avoid looking at the peripheral target, and instead to saccade to the opposite side as quickly as possible (Fig. 1b).

### Data analysis and statistics

Saccades were detected from the recorded eye positions using an automated velocity- and acceleration-based algorithm^31^, followed by visual inspection. Eye traces contaminated with blinks or gaze shift were first rejected. The eye position data was then low-pass filtered with a cut-off frequency of 250 Hz. Eye velocity and acceleration were estimated using the first-order and second-order derivatives of eye position signals, respectively. For saccade detection, two adaptive thresholds on eye velocity and acceleration were used for every trial. The thresholds were set to two standard deviations of eye velocity and acceleration. Saccades were detected when both eye velocity and acceleration passed the adaptive thresholds. Detected events with maximum velocity lower than ${20}^{^\circ }{s}^{-1}$ were rejected.

For each trial, we extracted parameters related to the corresponding saccade accuracy and speed. In the pro-saccade task, these included saccade latency and amplitude, while for the anti-saccade task, we quantified the latency of correct anti-saccades and error pro-saccades. We also measured the anti-saccade error rate which quantifies the percentage of trials with erroneous pro-saccades during the anti-saccade task. The anti-saccade error rate was used as a measure of inhibitory control failure.

For the pairwise comparisons (*i.e*. comparing one parameter of interest between two conditions), Generalized Linear Mixed Effect models (GLMEs)^32^ were used to measure the statistical significance of a given difference between the two conditions of interest with the changed factor between the two conditions as the fixed effect (DBS or L-dopa). Due to inter-individual differences in DBS and L-dopa effects, the participants’ identities were introduced as the random effect into the model. For example, in a comparison between the saccade latency in the OFF L-dopa – ON stimulation and OFF L-dopa – OFF stimulation conditions, the stimulation is introduced as the fixed effect, and the patients’ identities as the random effect.

We also used GLME model to run a mixed-effect two-way ANOVA across conditions for different eye movement parameters. For each measurement (e.g. reciprocal latencies and error rates), we used the ‘fitglme’ function in MATLAB to fit the GLME model to the data.

For GLME models to fit the data probability distribution, for all the measurements, the Akaike Information Criterion (AIC) was used to compare between different models with different output distributions: Normal, Gamma, and Inverse Gaussian. Based on these model comparisons, for the error rate and latencies, we used normal and inverse gamma distributions, respectively. Considering the skewness of saccade latency distribution, inverse gamma is a better fit for modeling the latency observations.

Furthermore, in the cases where the structure of the GLME model needed to be determined based on the data (e.g. adding or removing an interaction term, or a random-effect), we used AIC to compare between different model structures.

## Results

To measure the effect of STN DBS on anti-saccade behavior, we tested 10 PD patients (3 females, mean age $61.2\pm 2.97$; see Table 1) in four different conditions measuring both pro-saccade and anti-saccade behavior. Patients were tested ON or OFF L-dopa, while ON or OFF DBS.

Our tremor measurements indicate that both L-dopa and DBS have a significant effect in decreasing tremor amplitude (t-stat = 2.15, p-value = 0.03 for L-dopa, and t-stat = 3.04, p-value = 0.004 for DBS), confirming that effective STN DBS and L-dopa treatment was delivered during the oculomotor task. Moreover, the UPDRS-III evaluations show that the combination of DBS and L-dopa improved the participants’ motor performance (t-stat = 1.97, p-value = 0.057). To make the protocol shorter and prevent further fatigue in patients, we ran the UPDRS evaluations only in two phases of the test: OFF L-dopa – OFF stimulation and ON L-dopa – ON stimulation. Therefore, we are not able to report the effect of individual treatments and their interactions based on the UPDRS.

For the anti-saccade task, we compared four parameters across the four conditions: anti-saccade error rate (ER), correct anti-saccade latency (${T}_{a}^{AS}$), error pro-saccade latency in the anti-saccade task (${T}_{p}^{AS}$), and pro-saccade latency in the pro-saccade task (${T}_{p}^{PS}$).

### Anti-saccade error rate

**Figure 2:**
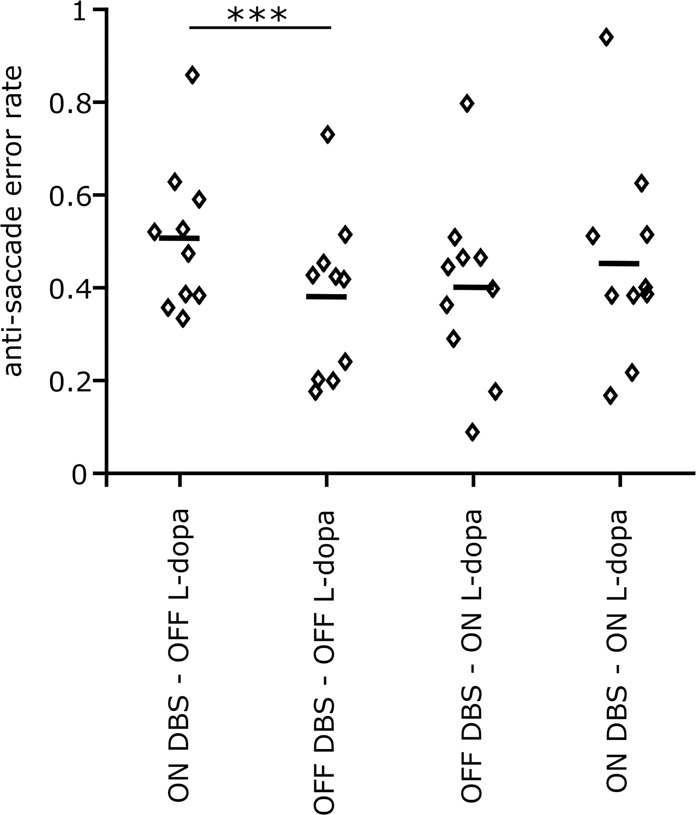
Anti-saccade error rate for different stimulation and L-dopa states. The small diamonds show the measured values for individual patients, and small horizontal lines show the median values. DBS increased the error rate during the Off L-dopa condition ($p < 0.001$). Based on our analysis of variance (two-way ANOVA), DBS also increases the error rate while on L-dopa, but not significantly ($p=0.15$). L-dopa does not have any significant effect on the error rate On or Off DBS (off DBS $p=0.32$, on DBS $p=0.24$). All statistics are driven from a fitted mixed-effect generalized linear model (GLME), where the L-dopa and DBS conditions were used as the fixed-effects and the patient’s identity as the random-effect.

The proportion of erroneous pro-saccades over all the trials is defined as the anti-saccade error rate. Figure 2 shows the error rates for individual patients (small diamonds) as well as the median error rate estimated across patients (small horizontal lines) for four different conditions. During the OFF L-dopa state, turning on STN DBS increases the anti-saccade error rate (t-stat = 5.64, p-value < 0.0001). During the ON L-dopa state, although STN DBS still increases the error rate, this change is smaller and not statistically significant (t-stat = 1.51, p-value = 0.15). There is no effect of L-dopa on the error rate, either ON or OFF DBS.

These observations suggest that STN DBS has a significant effect on anti-saccade error rate, and this effect can be modulated by medication state. To better quantify this interaction, we fitted a mixed-effect generalized linear model (GLME) to the error rate measurements across our cohort of subjects with L-dopa, DBS, and their interaction as the fixed-effects, and the patient identities as the random effect. The results are shown in Table 2. Similar to the pairwise comparison above, the GLME analysis indicates that STN DBS increases the error rate, and this effect is statistically significant. Moreover, the interaction analysis indicates that there is a negative interaction between L-dopa and DBS. The results show that the L-dopa decreases the effect of STN DBS on the error rate (t-stat = 1.97, p-value = 0.057). As a more rigorous statistical support for the DBS – L-dopa interaction, we compared two GLME models: one that included the interaction term ($ER=\,{\beta }_{0}+{\beta }_{dbs}.DBS+{\beta }_{ldopa}.Ldopa+\,{\beta }_{I}.DBS\ast Ldopa$) and one that did not ($ER=\,{\beta }_{0}+{\beta }_{dbs}.DBS+{\beta }_{ldopa}.Ldopa$). Model comparison techniques inform us about the superiority of one model over the other in terms of explaining the data. The results of this model comparison showed that our measured error rates were more reliably explained by the model that included the interaction term (Log-likelihood = 36.351, AIC = −50.7) compared to the one that did not (Log-likelihood = 34.65, AIC = −49.3). The likelihood ratio test showed that the mixed-effect model with the interaction was moderately more capable of describing our data (likelihood ration = 3.40).

**Table 2:** ANOVA results on anti-saccade error rate.

Name	Estimate	SE	t-stat	DF	p-value	lower	upper
DBS	0.127	0.029	4.2979	36	0.0001 (***)	0.067	0.1869
l-dopa	0.0208	0.029	0.71028	36	0.48211	−0.038592	0.080193
DBS * l-dopa	−0.0737	0.037	−1.9678	36	0.05683	−0.14964	0.002257

The observed interaction between DBS and L-dopa shows that while STN DBS increases the error rate in both L-dopa on and off conditions, this increase is larger while the patients are off L-dopa.

Next, we determined how STN DBS may alter the reaction times during both anti-saccade and pro-saccade tasks.

### Anti-saccade latency

There are two types of latencies in the anti-saccade task: the latency of correct anti-saccades (${T}_{a}^{AS}$), and the latency of the erroneous pro-saccade (${T}_{p}^{AS}$). On average, the latency of pro-saccades is shorter than that of the correct anti-saccades, possibly due to further processing needed to generate anti-saccades^20^, generally referred to as the “anti-saccade cost”. Here, we measure the changes in these two types of latencies caused by changes related to being ON or OFF L-dopa, and ON or OFF DBS.

**Figure 3:**
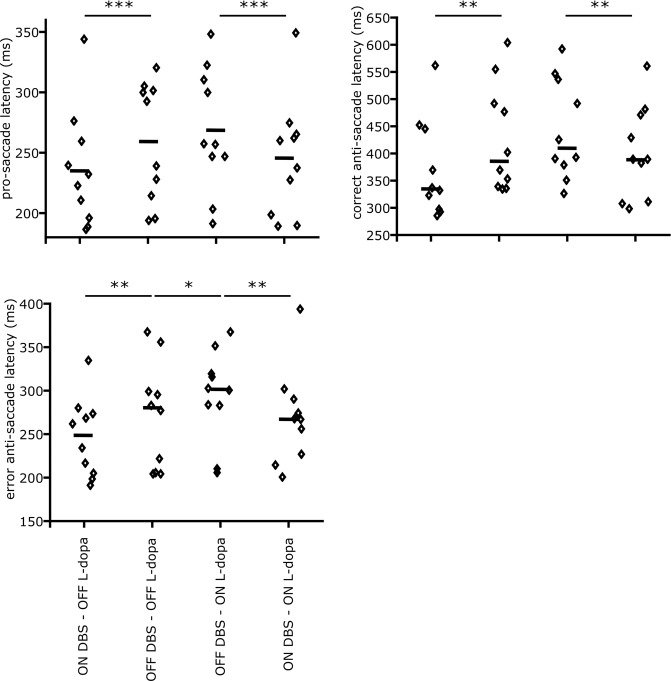
Anti-saccade and pro-saccade latencies for different stimulation and L-dopa states. The small diamonds show the measured values for individual patients, and small horizontal lines show the median values. (**a**) The effect of DBS and L-dopa on the pro-saccade latency. Based on our analysis of variance (two-way ANOVA), DBS decreases the pro-saccade latency in both L-dopa conditions ($p < 0.001$). L-dopa increases pro-saccade latency, but it is marginally significance ($p=0.062$). *(***b***)* The effect of DBS and L-dopa on the correct anti-saccade latency. DBS decreases the correct anti-saccade latency in both L-dopa conditions ($p=0.011$). The effect of L-dopa on the correct anti-saccade latency is not statistically significant, but follows the same pattern as in the pro-saccade latency. (**c***)* The effect of DBS and L-dopa on the erroneous pro-saccade latency in the anti-saccade task. All statistics are driven from a fitted mixed-effect generalized linear model (GLME), where the L-dopa and DBS conditions were used as the fixed-effects and the patients’ identity as the random-effect.

In Fig. 3b, the measured latencies for individual patients as well as their median values for the four conditions are shown. Our results show that the DBS *decreases* the latency for both error pro-saccades (t-stat = 3.12. p-value = 0.006), and correct anti-saccades (t-stat = 2.82, p-value = 0.011). However, L-dopa only *increases* the pro-saccade latency (t-stat = 2.34, p-value = 0.03), and has no significant effect on anti-saccade latency (t-stat = 1.55, p-value = 0.14). Our interaction analysis also shows that there is no interaction between the DBS and L-dopa effects either for pro-saccade (t-stat = 0.62, p-value = 0.54) or anti-saccade latencies (t-stat = 1.3, p-value = 0.2).

In summary, STN DBS *decreases* the correct pro-saccade and erroneous pro-saccade latencies, while L-dopa *increases* only the pro-saccade latency.

**Figure 4:**
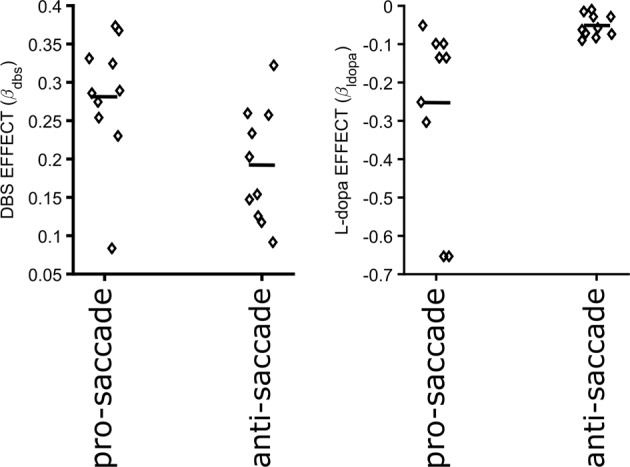
DBS (right) and L-dopa (left) effects on pro-saccade and anti-saccade latencies. Small diamonds show the estimated effects for individual patients ($fixed\,effect+random\,effect$ from the GLME models), and the small horizontal lines show the average effect across subjects ($fixed\,effect$ from the GLME model). Positive and negative values show decrease and increase in the saccade latencies, respectively. The estimated effect sizes show that DBS decreases both pro-saccade and anti-saccade latencies. The effect is larger on pro-saccades than anti-saccades. L-dopa causes an increase in pro-saccade latency, but has a close to zero effect on the anti-saccade latency.

To quantify the effect of DBS and L-dopa across subjects, we fitted a GLME to our measured latencies where the DBS and L-dopa conditions were set as the fixed effects while the patients’ identity were set as the random effect (e.g. ${T}_{a}^{AS}=\,g({\beta }_{0}+{\beta }_{dbs}DBS+{\beta }_{ldopa}Ldopa)$). ${\beta }_{dbs}$ and ${\beta }_{ldopa}$, the estimated coefficients of DBS and L-dopa factors in the model, quantify the effect of each intervention on the behavioral measurements. We repeated the same analysis for erroneous pro-saccade and correct anti-saccade latencies. In Fig. 4, the estimated effects for every subject ($fixed\,effect+random\,effect$; small diamonds) as well as the average estimated effects across subjects ($fixed\,effect$; small horizontal lines) are shown. As shown in Fig. 4, The estimated ${\beta }_{dbs}$ and ${\beta }_{ldopa}$ were both larger for pro-saccade than anti-saccade latencies which reflected the larger effect of both interventions on pro-saccade latency.

We next consider the effects of interventions on the saccade latency in the pro-saccade task (${T}_{p}^{PS}$).

### Pro-saccade latency

Figure 3a shows the changes in the pro-saccade latency (${T}_{p}^{PS}$) as a function of L-dopa and DBS states. Reproducing previous observations^33^, our results show that STN DBS decreases the pro-saccade latency (t-stat = 3.7, p-value = 0.0007), while L-dopa increases it, though the effect of L-dopa is not significant (t-stat = 1.92, p-value = 0.062).

On average, the latency changes that we showed here in the pro-saccade task follow the changes that we reported in previous section for the error pro-saccades in the anti-saccade task (Fig. 3).

### DBS effect on pro-saccade vs. anti-saccade latencies

**Figure 5:**
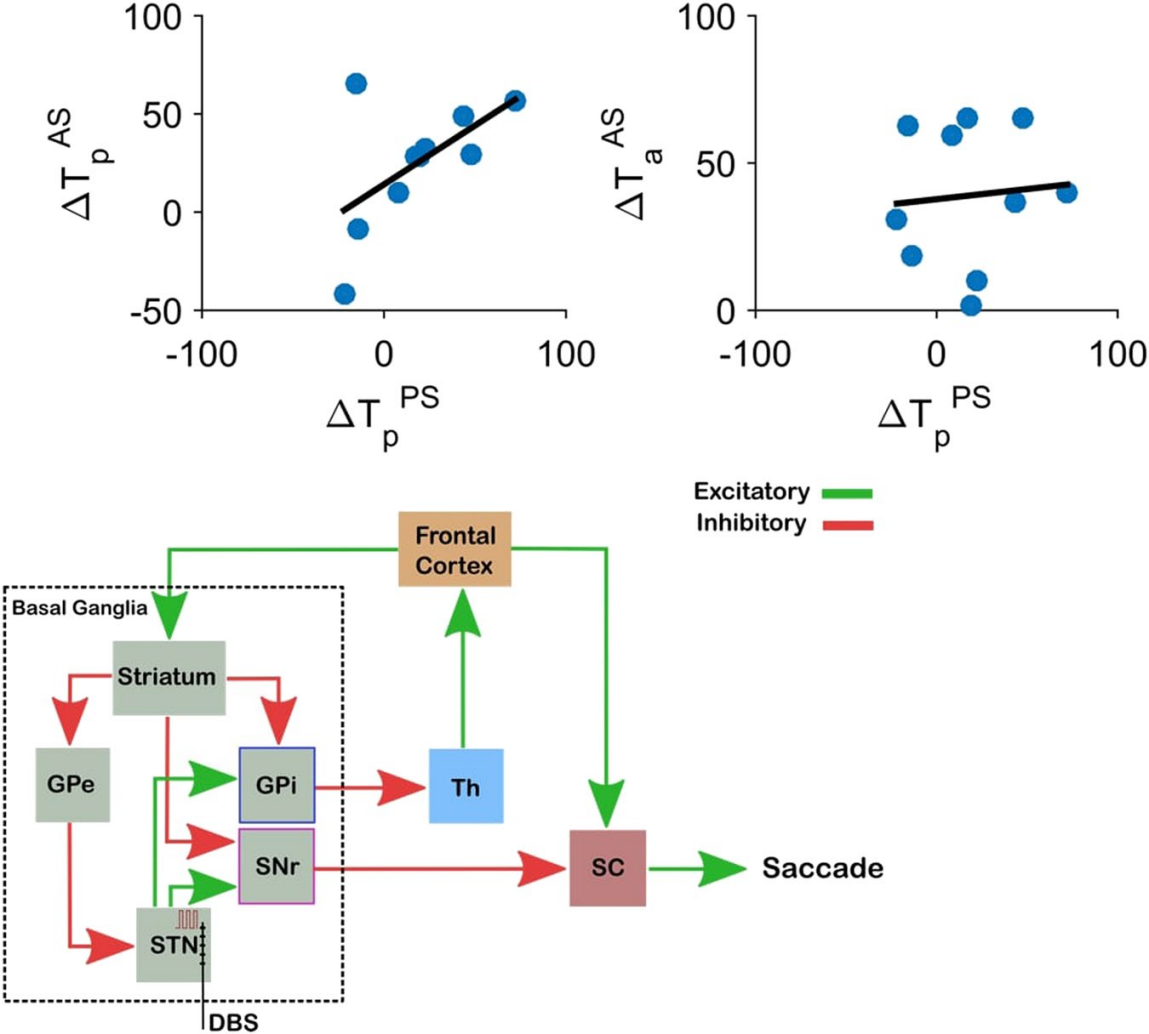
The effect of DBS on pro- vs. anti-saccade latencies. Blue circles show the change in latencies for pro- and anti-saccade in each individual participant. The dark line is the fitter linear model to the data. (Top-left) There is a strong positive correlation between the change in the pro-saccade latencies in the both pro-saccade and the anti-saccade tasks ($r=0.6$, $p < 0.001$). (Top-right) The changes in the pro-saccade latency and the anti-saccade latency caused by DBS are not correlated ($r=0.07$, $p=0.12$). (Bottom) A schematic of the oculomotor network that involves the basal ganglia and relevant projections. Excitatory (green) and inhibitory (red) projections are shown. SNr and GPi, the two output nuclei of the basal ganglia, project to SC and thalamus (Th). STN DBS can influence saccades through either the GPi-Th-Frontal Cortex pathway, or the SNr-SC pathway. Lower brainstem, cerebellar and extra-frontal cortical oculomotor pathways are not illustrated.

In Fig. 5a, we compare the effect of DBS on pro-saccade latencies in the pro-saccade (horizontal axis) and the anti-saccade tasks (vertical axis) within individual subjects. Each circle corresponds to the changes in latencies for each subject. The results show a strong correlation ($r=0.6$) between these two effects across subjects.

On the other hand, the correlation did not hold when we compared the effect of DBS on pro-saccade and anti-saccade latencies ($r=0.07$) (Fig. 5b). The same observation was made with measured pro-saccade latencies in either the pro-saccade task or the anti-saccade. In each subject, the effect of DBS on the pro-saccade latency is therefore not predictive of its effect on the anti-saccade latency.

This observation supports the idea that pro- and anti-saccades are controlled by neural circuits that are at least partially independent (see discussion). As shown in this and previous sections, both DBS and L-dopa can have distinct effects on these independent components, and the effect sizes change across subjects.

## Discussion

In the anti-saccade task, two main actions compete to access oculomotor resources: looking towards the target (pro-saccade), and looking away from the target (anti-saccade). Despite overlapping neural pathways^34^, these two inter-related actions are controlled by partially independent circuits^35^. The Superior Colliculus (SC) along with Frontal Eye Fields (FEF) are the main nodes in the oculomotor circuitry that directly influence the premotor circuit for saccade initiation. Therefore, in both pro-saccade and anti-saccade behaviors, the activity of SC and FEF drive the eye movement. However, the ability to suppress a reflexive pro-saccade and perform an anti-saccade reflects successful inhibitory control that is mainly controlled by the frontal and prefrontal areas^15^.

In Parkinson’s disease, deficiency in inhibitory control has been reported while patients perform anti-saccade^22,36^ and other similar experimental tasks^37^. Compared to healthy controls, Parkinson’s disease (PD) patients experience more difficulty suppressing the pre-potent reflexive response (e.g. pro-saccade), that allows a voluntary action (e.g. anti-saccade). Although STN DBS and L-dopa successfully reduce many motor symptoms of PD, their effects on inhibitory control is uncertain. Here we used the anti-saccade task to measure the effects of STN DBS and dopaminergic medication on inhibitory control in PD patients. The present work demonstrates that L-dopa prolongs only pro-saccade latency, and not anti-saccades. The increase in pro-saccade latency is in keeping with previous work^38^, but the effect of L-dopa and dopamine agonists on anti-saccades (error rate and latency) remains controversial^39,40^. The variability of dopaminergic medication dose across patients and heterogeneity of the disease might underlie this uncertainty. Importantly, our results show that the effect of STN DBS on anti-saccade error rate depends on the L-dopa state of the patients. We elaborate more on our findings in the following sections.

### The effect of STN DBS on saccades

The subthalamic nucleus has anatomical connections with both low-level oculomotor nuclei through nigro-collicular projections^41,42^ and high-level frontal and prefrontal areas^4,41,42^, and therefore may be an important hub regulating behaviors related to visuo-motor action selection. The precise role of the subthalamic nucleus in different aspects of oculomotor performance in human, especially in inhibitory control, is uncertain, and may be explored by taking advantage of subthalamic stimulators inserted for relief of motor symptoms of PD.

Our results show that STN DBS decreases both pro-saccade and anti-saccade latencies, and increases anti-saccade error rate. On average, our data indicate a larger effect of STN DBS on pro-saccade than anti-saccade latencies (Fig. 4 – left). Previous attempts at examining the effect of STN DBS on anti-saccade behavior have produced variable observations. Of several studies that specifically addresses effects of STN DBS of anti-saccades^28–30^, only one evaluated patients in their off-L-dopa condition, reporting results similar to the present study^30^. Two other studies^28,29^ tested patients while they were on their regular dose of L-dopa, and reported no significant effect of STN DBS on anti-saccade performance. In the present work, we suggest that the L-dopa state can explain the discrepancies in the previous studies. Based on our results, STN DBS increases the anti-saccade error rate significantly only when patients are Off L-dopa (Fig. 4 – right). In the On L-dopa condition, STN DBS still increases the error rate but not significantly, which suggests that the effect of STN DBS is mitigated by the L-dopa On state. This interpretation of our data is potentially limited by the design of our experiment. We further elaborate on this limitation at the end of this section.

Unlike their effects on the eye movements, both L-dopa and DBS improve tremors in patients. This apparent conflict is not surprising given the distinct circuitries underlying oculomotor behavior and tremor within the basal ganglia and their targets^43^.

Previous electrophysiology, neuroimaging, and computational modeling studies support the role of the STN in cognitive-associational behavior^44^. In particular, more recent electrophysiology studies in animals and humans implicate STN in inhibitory control^45–48^. The role of the STN in suppressing prepotent responses in inhibitory control tasks (e.g. stop-signal task, Stroop task, and countermanding saccade), has been shown in neuroimaging^46^, electrophysiology^45,49^, and lesional^50^ studies. In a stop-signal task, for example, Schmidt et al. (2013) showed that STN neurons in rats exhibit short latency responses to the ‘Stop’ signal, which encodes the necessity of canceling a prepared ‘Go’ response. Similar observations were reported in humans using fMRI^46^. These results are consistent with our observation that STN DBS interferes with inhibitory control, and increases the anti-saccade error rate in PD patients.

Moreover, recent studies highlight the importance of beta oscillations in STN and its context-dependent desynchronization for successful motor and cognitive performance^48,51^. In PD, pathological hyperactivity^52,53^ and exaggerated beta synchronization affect the normal function of the STN. As suggested by the rate model of direct/indirect pathways of the basal ganglia^54^, the STN has an overall inhibitory effect on action generation. Under healthy physiological states, firing of STN neurons selectively suppresses the activity of frontal areas which in turn prolongs action generation. The appropriate level of STN activity is therefore required for generation of contextually appropriate actions, and suppressing inappropriate ones. In PD, the pathological over-expression of synchronized bursts of STN activity leads to abnormally delayed and contextually inappropriate saccades. This may explain the slowness of saccades as well as higher error rate of anti-saccades in PD^10^. However, depending on the disease stage, the basal ganglia circuitry is variably deficient. For example, pathological activity in STN correlates with the symptom severity^55^. Therefore, the residual functionality may still allow some functional control of oculomotor and other associative behaviors. More pronounced interference with abnormal STN activity may then result in a more detrimental effect on cognitive control. Our results support this hypothesis. Increased inhibition of STN function with DBS may impair a residual control mechanism that allows anti-saccade behavior. Since the STN has an inhibitory effect on saccade-related brain regions (e.g. superior colliculus and frontal eye field), eliminating it from the oculomotor circuit facilitates saccade generation regardless of their importance in the task, which can explain the decreased latencies of both pro- and anti-saccades. Moreover, pro-saccades, since they generally involve a faster process compared to an anti-saccade, would be expected to reach the decision threshold sooner. This may explain the higher error rate that we observe in the ON DBS condition compared to OFF DBS.

### STN in oculomotor circuitry

During a saccade task, previous electrophysiology studies have shown that neuronal firing rates in frontal eye field and superior colliculus ramp up prior to saccade generation^20,56^. The firing rates that correspond to different possible actions in these areas (e.g. pro-saccade vs. anti-saccade) increase to a hypothetical threshold, and the action that first reaches this threshold is selected. The slope of this increase in firing rate determines how fast one commits an eye movement. Therefore, a control mechanism on this slope can favor one action over another based on the context.

Particularly, since the pro-saccade is the incorrect behavior in the anti-saccade task, its corresponding neuronal activity in frontal areas should be controlled to increase less rapidly compared to the anti-saccade. As part of the basal ganglia indirect pathway, STN’s activity can decelerate the increase of firing rates for an action (e.g. pro-saccade in our example) when necessary given the contextual information. Based on the present results, we suggest that STN plays an important role in this coordination process. A mechanistic model such as drift diffusion model^57^ or LATER^58,59^ can potentially simulate this process. However, some aspects of our experimental observations, as described below, cannot be reproduced with the current version of these models (for example, see Fig. 5).

As suggested by Jantz et al.^60^, the suppressive effect that the STN-SNr pathway can impose on saccades by inhibiting the SC, is essential for goal-directed eye movements, including anti-saccade behavior. In general, anti-saccade behavior is slower than pro-saccades. The additional time required for planning and executing the anti-saccade behavior is granted through suppressing reflexive pro-saccades by the STN-SNr inhibitory effect. Our results suggest that STN DBS interferes with the normal function of the STN-SNr-SC pathway, interfering with abnormal goal-directed behavior. This abnormality is captured by the increased anti-saccade error rate in the DBS ON condition.

Our correlation analysis shows that the DBS effect on pro-saccades in individual subjects does not predict the effect on their anti-saccades (Fig. 5, top). These observations support the idea that anti-saccade and pro-saccade behaviors are partially controlled by independent circuits, with frontal and prefrontal areas being more involved in anti-saccade behavior^35^, and the pro-saccades more controlled by the superior colliculus. In general, our results indicate that DBS and L-dopa can differentially affect pro-saccade and anti-saccade behaviors. This is in contrast with the assumption in previously proposed models^59^.

A dual pathway model (Fig. 5, bottom) may explain previous observations on differential effects of pallidal and subthalamic DBS on different saccade behaviors^29^. In their results, Antoniades et al. showed that for patients on L-dopa medication, STN DBS does not significantly alter anti-saccade error rate. However, DBS of the internal Globus Pallidus (GPi) reduces the error rate. In the present study, STN DBS significantly increased anti-saccade error rate especially when patients were off L-dopa. Thus both GPi and STN are important elements of the circuitry modulating anti-saccade behavior. Ultimately, both nuclei influence relevant frontal areas, including the supplementary motor and dorsolateral prefrontal cortices implicated in attention. The STN, via inputs to the SNr^61^, also provides input to the superior colliculus^62^ and thalamic areas^63^ projecting to frontal eye fields. These differences in circuitry may help explain the uncorrelated effects of STN DBS on both pro-saccades and anti-saccades (Fig. 5) seen in the present study. These circuit differences may also underlie differences in anti-saccade behavior in response to GPi or STN DBS.

Finally, effects of pallidal or STN DBS on anti-saccade error rate are of interest in the context of behavioral impulsivity. PD patients can show impulsivity, and this behavior may be exacerbated by STN or GPi DBS or dopaminergic therapy^64^. Since anti-saccade error rates may correlate with impulsivity in PD and other neurodegenerative disorders^65^, oculomotor behavior could be developed as a readily measurable surrogate for impulsivity, and allow appropriate titration of DBS, L-Dopa or dopamine agonist therapy.

### Limitations

An important limitation of the current study is the fixed, non-randomized sequence of task conditions in our protocol. As a result, confounding factors such as fatigue or task familiarity, could potentially influence our analysis. We followed this specific sequence of conditions to be able to test patients in all four DBS/L-dopa conditions within one day. For example, the ON L-dopa condition could not be tested before the OFF L-dopa, since OFF L-dopa condition patients had to stop the medication for at least 12 hours. We also avoided a between-group design, by splitting patients into different group conditions, to reduce the possible effect of inter-individual variabilities that could drastically decrease our statistical power. However, it is possible that at least part of our observed behavioral changes (e.g. in the anti-saccade error rate or latencies) were due to confounding factors such as fatigue or familiarity. A complete dismissal of effect of these confounding factors would be possible only under a randomized experiment design which, as explained above, was not feasible given the main aim of this study. Future studies should explicitly address this limitation.

## Summary

Our results support the crucial role of STN in the anti-saccade task. We draw three important conclusions based on our results: first, pro-saccades and anti-saccades are driven by partially independent mechanisms, and DBS and L-dopa can modulate the two systems independently. Second, eliminating STN from the saccade circuitry decreases the reaction times for both pro- and anti-saccades. As pro-saccades are mainly generated faster than anti-saccades, the DBS effect is relatively more pronounced on pro-saccades than anti-saccades, and the action facilitation caused by STN DBS leads to higher anti-saccade error rate. Third, even though STN activity is abnormal in PD, the residual functionality of STN is needed for complex cognitive behaviors. Marked reduction of STN activity by DBS shuts down this functionality, and manifests as a higher anti-saccade error rate.
